# Supplementary material for: Metallothionein Family Proteins as Regulators of Zinc Ions Synergistically Enhance the Anticancer Effect of Cannabidiol in Human Colorectal Cancer Cells
Source: Int J Mol Sci. 2023 Nov 22;24(23):16621. doi: 10.3390/ijms242316621 (PMC10705991; doi:10.3390/ijms242316621)
Supplement: Supplementary file 1 [file ijms-24-16621-s001.zip › ijms-2712904-supplementary.pdf]

Supplementary Figures.

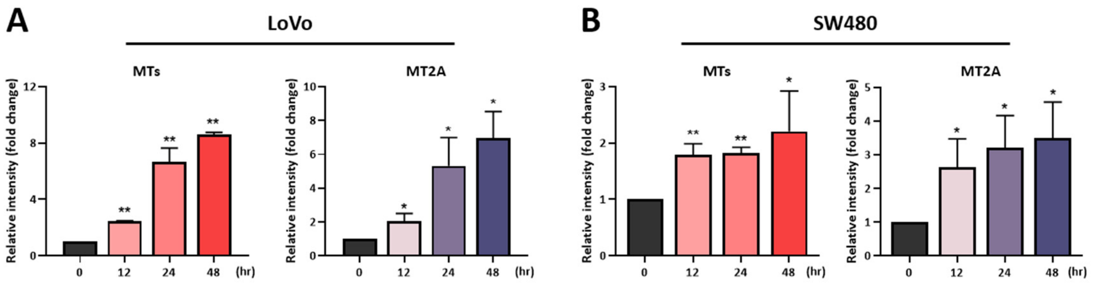

Supplementary Figure S1. Densitometric analysis on Western blot data of MT expression by CBD treatment. Western Blot data were quantified by densitometry using ImageJ v1.54d. The graphs of metallothionein protein levels were shown in (A) LoVo and (B) SW480 cells. Statistical significance was considered with p -value (*, $p \leq 0.05$; **, $p \leq 0.01$).

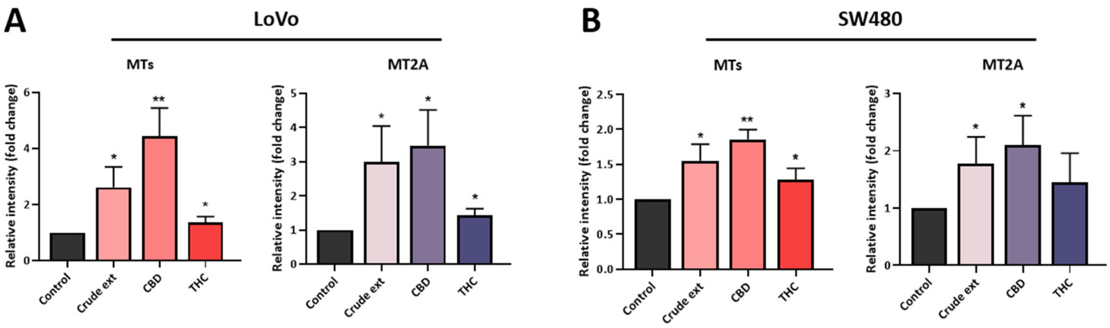

Supplementary Figure S2. Densitometric analysis on Western blot data of MT expression by cannabinoid treatment. Western Blot data were quantified by densitometry using ImageJ v1.54d. The graphs of metallothionein protein levels were shown in (A) LoVo and (B) SW480 cells. Statistical significance was considered with p-value (*, $p \leq 0.05$; **, $p \leq 0.01$).

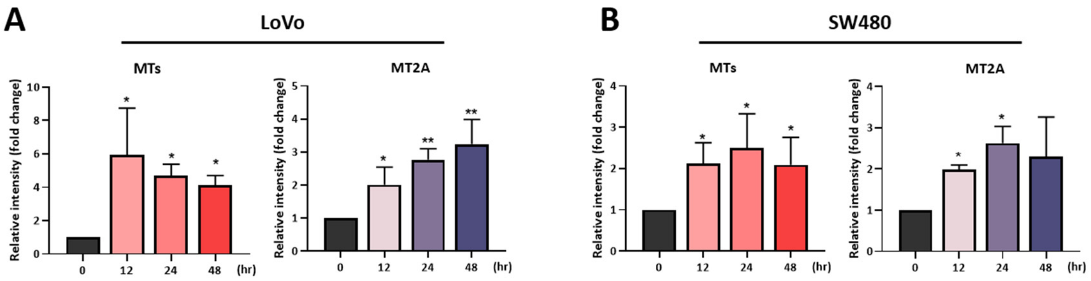

Supplementary Figure S3. Densitometric analysis on Western blot data of MT expression by zinc ion treatment. Western Blot data were quantified by densitometry using ImageJ v1.54d. The graphs of metallothionein protein levels were shown in (A) LoVo and (B) SW480 cells. Statistical significance was considered with p -value (*, $p \leq 0.05$; **, $p \leq 0.01$).

Supplementary Table S1. List of primers used for qPCR

Gene	Sequence (5'→3')
**MT1F**	**Forward: CTG GTG TCT CCT GCA CCT G**
	**Reverse: CAG CAG CTG CAC TTC TCT GA**
**MT1G**	**Forward: AAA GGG GCA TCG GAG AAG TG**
	**Reverse: GCA AAG GGG TCA AGA TTG TAG C**
**MT1H**	**Forward: TGG ACC CCA ACT GCT CCT**
	**Reverse: ACT TCT CTG ACG CCC CTT TG**
**MT1M**	**Forward: TCG AAA TGG ACC CCA ACT GCT**
	**Reverse: TTC CCA CAT CAG GCA CAG CA**
**MT1X**	**Forward: CTG CTT CTC CTT GCC TCG AA**
	**Reverse: TGT CTG ACG TCC CTT TGC AG**
**MT2A**	**Forward: GGA TCC CAA CTG CTC CTC CTG C**
	**Reverse: CTT GTC CGA CGC CCC TTT**
**Beta-actin**	**Forward: CCA CCA TGR ACC CTG GCA TT**
	**Reverse: CGG ACT CGT CAT ACT CCT GC**
